# Zinc finger factor 521 enhances adipogenic differentiation of mouse multipotent cells and human bone marrow mesenchymal stem cells

**DOI:** 10.18632/oncotarget.3900

**Published:** 2015-05-06

**Authors:** Kuo-Yun Tseng, Shankung Lin

**Affiliations:** ^1^ Institute of Cellular and System Medicine, National Health Research Institutes, Miaoli, Taiwan, Republic of China; ^2^ Graduate Institute of Basic Medical Science, China Medical University, Taichung, Taiwan, Republic of China

**Keywords:** aging, marrow MSC, age-related bone loss, osteoporosis, adipogenesis

## Abstract

Previously, we found that ZNF521 expression was up-regulated with advancing age in human bone marrow mesenchymal stem cells (bmMSCs). Here, we investigated the regulatory role of ZNF521 in the differentiation of mouse C3H10T1/2 cells and human bmMSCs. Our data show that ZNF521 overexpression repressed osteoblastic differentiation of C3H10T1/2 cells, accompanied by a decrease in Runx2 expression and an increase in PPARγ2 expression. In contrast, ZNF521 overexpression enhanced adipogenic differentiation of C3H10T1/2 cells, concomitant with increased expression of PPARγ2, aP2, adiponectin and C/EBPδ. Chromatin immunoprecipitation followed by quantitative PCR analyses and luciferase reporter assays suggested that ZNF521 overexpression enhances PPARγ2 expression at the transcriptional level. The enhancing effect of ZNF521 overexpression on the adipogenic differentiation of C3H10T1/2 cells was also observed *ex vivo*. Finally, similar to those noted in C3H10T1/2 cells, ZNF521 overexpression in human bmMSCs was found to promote adipogenic differentiation *in vitro* and *ex vivo*, but repressed osteoblastic differentiation *in vitro*. ZNF521 knockdown significantly repressed adipogenic differentiation *in vitro* and *ex vivo*, but promoted osteoblastic differentiation *in vitro*. We propose that ZNF521 can function as a repressor of osteoblastic differentiation of bmMSCs while promoting adipogenesis, and that elevated ZNF521 expression might play a role in the age-related bone loss.

## INTRODUCTION

It is well documented that bone mass decreases and bone fragility increases with age in both men and women, and that such an age-related bone loss is closely related to the pathogenesis of osteoporosis [1 and references therein]. Although an increase in bone resorption rate associated with menopause is the primary cause of low bone mass in postmenopausal women, a decline in bone formation rate is a critical contributor to postmenopausal- and age-related bone loss. Therefore, elucidating how bone formation rate decreases with age may provide insight into the mechanisms underlying the age-related bone loss, and may benefit the development of effective strategies for treating osteoporosis.

In bone marrow, bone formation is carried out by osteoblast. It is currently unclear if aging impairs the bone-forming capability of osteoblast; however, it has been reported that there are fewer osteoblasts in aging bones than in young bones, and that the decrease of bone mass and osteoblast numbers in aging bones is accompanied by the increase of fat deposits and adipocyte numbers [2–4]. Moreover, comparison of the regenerated bone marrows of young and old rats after marrow ablation shows that the regenerated bone marrows of old rats are filled with numerous fat deposits far more than that observed in the bones of young rats, and there is a concurrent decrease in osteoblast numbers in marrows of old bones [5]. These findings indicate that osteoblast production decreases with aging, and that bone marrows become more adipogenic than osteoblastogenic in aging bones. Given the fact that osteoblasts and adipocytes are originated from bone marrow mesenchymal stem cells (bmMSCs), the regulatory program that works as a function of age to suppress osteoblatic lineage but to promote adipogenic lineage of bmMSCs might play an important role in the age-related decline in bone formation rate.

To date, several transcription factors have been shown to participate in the lineage differentiation of bmMSCs. Factors such as runt-related transfection factor 2 (Runx2) and peroxisome proliferator-activated receptor-γ (PPARγ) are key factors that determine the lineage commitment; Runx2 is for osteoblastic lineage [6, 7], whereas PPARγ is for adipogenic lineage [8, 9]. PPARγ is expressed in two isoforms, PPARγ1 and PPARγ2, with PPARγ2 as the major regulator in adipocyte formation [10]. Like the mutually exclusive pattern between the adipogenic and osteoblastic differentiation of bmMSCs, the activity of PPARγ and Runx2 is reciprocally regulated in bmMSCs to modulate MSC differentiation. A factor called TAZ has been found to serve as a regulator [11]. Likewise, Moerman et al. have reported that aging increases PPARγ expression and activates the adipogenic program in mouse bmMSCs, which is accompanied by a decrease in Runx2 expression and the commitment to osteoblastic lineage [12]. These findings suggest the presence of as yet undocumented factors that regulate the switch in the lineage commitment of bmMSCs as a function of age (or aging).

We have recently analyzed the gene expression profiles of human bmMSCs derived from donors of varying ages, and identified a list of potential age-associated genes [13]. Among them, *ZNF521* is one of the genes with expression up-regulated with donor age. *ZNF521* encodes the transcription factor zinc finger factor 521 (ZNF521) which shares 97% homology with mouse homolog zinc finger protein 521 (Zfp521). Human ZNF521 and mouse Zfp521 each consists 30 C2H2 Kruppel-like zinc finger motifs, with molecular weight around 148 kDa. The functions of ZNF521 have not been well characterized. While its potential involvement in regulating osteogenesis and adipogenesis has been suggested [14, 15], the question as to whether ZNF521 promotes adipogenesis or osteogenesis requires further investigation.

Here, we investigate the role of ZNF521 in the adipogenic and osteoblastic differentiation of mouse multipotent C3H10T1/2 cells and human bmMSCs. Our data suggest that ZNF521 acts as an enhancer of adipogenic differentiation but as a repressor of osteoblastic differentiation in both types of cells, supporting the notion that increased ZNF521 expression with age might contribute to age-related bone loss.

## RESULTS

### ZNF521 overexpression repressed osteoblastic differentiation of C3H10T1/2 cells

**Figure 1:**
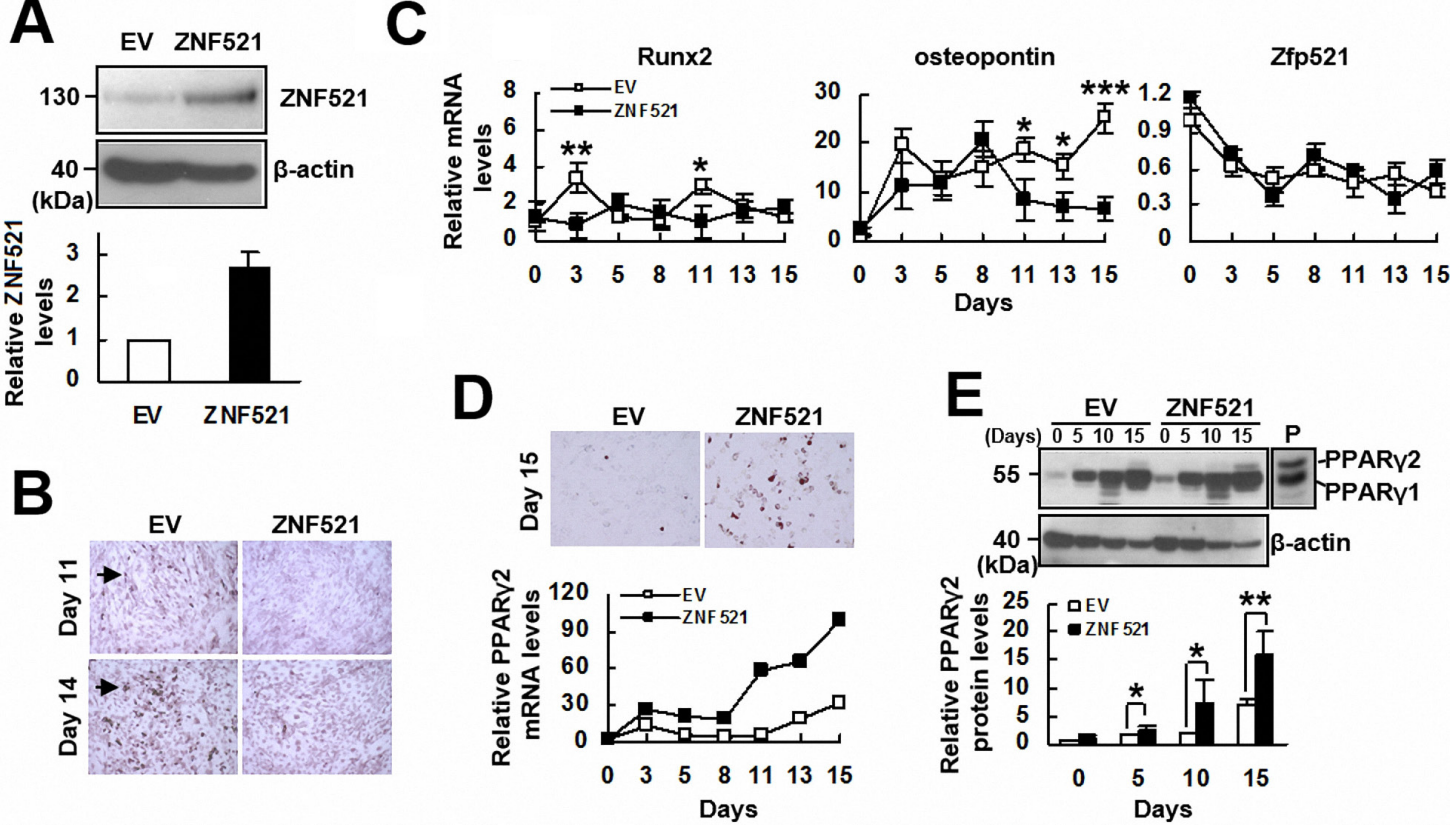
The effect of ZNF521 overexpression on the osteoblastic differentiation of C3H10T1/2 cells **A.** Western blot analyses. Relative ZNF521 levels were calculated by comparing the normalized ZNF521 plus Zfp521 signals of C3H-ZNF521 (ZNF521) cells to the normalized Zfp521 signals of C3H-EV (EV) control (to which *a* value of 1 was assigned). Data represent the means ± S.D. from three preparations. **B.** Osteoblastic induction. Confluent EV and ZNF521 cultures were induced to undergo osteoblastic differentiation, and stained with Alizarin Red S. Stained cells were indicated by arrowhead. **C.** RT-qPCR analyses. EV and ZNF521 cells undergoing osteoblastic differentiation were harvested at the days as indicated for the measurement of Zfp521, Runx2, osteopontin, and β-actin mRNAs. Relative expression levels were calculated in relation to the EV control at day 0 (to which *a* value of 1 was assigned). Data represent the means ± S.D. from three experiments. *, *P* < 0.05; **, *P* < 0.01; ***, *P* < 0.005 versus corresponding control. **D.** Oil Red O staining and RT-qPCR analyses. EV and ZNF521 cells undergoing osteoblastic differentiation were stained with Oil Red O. Representative images are shown. Cells were harvested at the days as indicated, and the levels of PPARγ2 mRNA were calculated in relation to the EV control at day 0 (to which *a* value of 1 was assigned). **E.** Western blot analyses. PPARγ2 levels of EV and ZNF521 cells were compared to that of EV control at day 0 (to which *a* value of 1 was assigned). Cell lysates of 3T3-L1 cells were used as a positive control (P) *, *P* < 0.05; **, *P* < 0.001.

To assess if ZNF521 participated in regulating the lineage differentiation of multipotent cells, we first examined if ZNF521 regulated the osteoblastic differentiation of C3H10T1/2 cells. We transfected C3H10T1/2 cells either with a plasmid harboring *ZNF521* cDNA or with an empty vector to establish C3H-ZNF521 and C3H-EV cells, respectively. Western blot analyses were performed to show the overexpression of ZNF521 (Figure 1A). We induced C3H-EV and C3H-ZNF521 cells to undergo osteoblastic differentiation, and found that ZNF521 overexpression inhibited osteoblastic differentiation, as evidenced by the Alizarin Red S staining 11 and 14 days post-induction (Figure 1B). We also harvested cells 0, 3, 5, 8, 11, 13, and 15 days post-induction to examine the expression of Runx2, osteopontin, and endogenous Zfp521 mRNAs. RT-qPCR analyses showed that induction of osteoblastic differentiation resulted in higher Runx2 expression in C3H-EV cells than in C3H-ZNF521 cells around the third and the eleventh day post-induction, and that ZNF521 overexpression showed the trend to delay and attenuate the induction of Runx2 expression (Figure 1C). Osteopontin expression was increased in both groups of cells; however, its expression continued to increase in C3H-EV cells but dropped in C3H-ZNF521 cells around the final five days of experiments. Endogenous Zfp521 mRNA levels decreased in a similar kinetics in both groups. On the other hand, we observed lipid droplet formation in C3H-EV and C3H-ZNF521 cells undergoing osteoblastic differentiation. Oil Red O staining performed 15 days post-induction showed more lipid droplet formation in C3H-ZNF521 cells than in C3H-EV cells, and RT-qPCR analyses also suggested more PPARγ2 expression in C3H-ZNF521 cells than in C3H-EV cells (Figure 1D). Subsequently, we performed Western blot analyses to examine PPARγ2 protein levels in C3H-EV and C3H-ZNF521 cells harvested 0, 5, 10, and 15 days post-induction. The results showed that induction of osteoblastic differentiation increased PPARγ2 expression in C3H-EV and C3H-ZNF521 cells, and that C3H-ZNF521 cells expressed more PPARγ2 than C3H-EV cells did during differentiation (Figure 1E). Taken together, our data show that ZNF521 overexpression inhibited osteoblastic differentiation of C3H10T1/2 cells, which was accompanied by a decrease of Runx2 expression and an increase of PPARγ2 expression.

### ZNF521 overexpression promoted adipogenic differentiation of C3H10T1/2 cells

**Figure 2:**
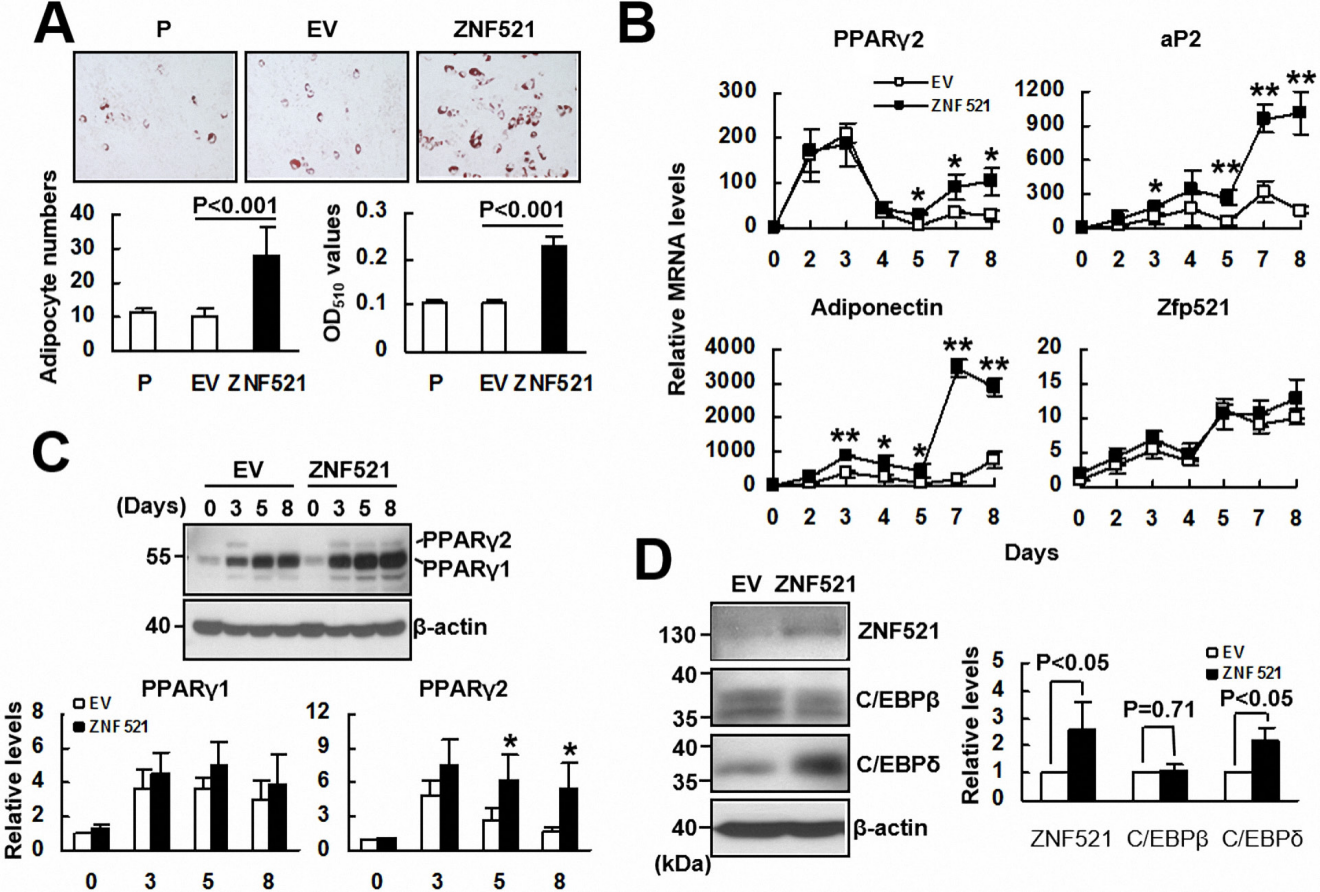
The effect of ZNF521 overexpression to the adipogenic differentiation of C3H10T1/2 cells **A.** Adipogenic induction. Confluent cultures of C3H-EV (EV) and C3H-ZNF521 (ZNF521) cells were induced to undergo adipogenic differentiation, and stained with Oil Red O. Representative images are shown. Stained cells were counted in 6 fields under a 200X high-power field. Each value represents the mean ± S.D. of 18 counts (left). Then, Oil Red O stains were solubilized and measured at 510 nm (right). **B.** RT-qPCR analyses. EV and ZNF521 cells were harvested at the days as indicated during differentiation, and examined for the levels of Zfp521, PPARγ2, adiponectin, and aP2 mRNAs in relation to that of EV control at day 0 (to which *a* value of 1 was assigned). Data represent the means ± S.D. from three experiments. *, *P* < 0.005; **, *P* < 0.0005 versus corresponding control. **C.** Western blot analyses. EV and ZNF521 cells harvested during differentiation were examined for the PPARγ1 and PPARγ2 protein levels in relation to that of EV control at day 0 (to which *a* value of 1 was assigned). Data represent the means ± S.D. from three experiments. ^#^, *P* = 0.11; *, *P* < 0.05. **D.** Western blot analyses. ZNF521, C/EBPβ, C/EBPδ, and β-actin protein levels of ZNF521 cells cultured without inducing differentiation were compared respectively to that of EV control (to which *a* value of 1 was assigned). Data represent the means ± S.D. from three experiments.

Since ZNF521 overexpression repressed osteoblastic differentiation, we examined if ZNF521 overexpression promoted osteoblastic differentiation of C3H10T1/2 cells. We induced parental C3H10T1/2, C3H-EV and C3H-ZNF521 cells to undergo adipogenic differentiation, and examined the accumulation of lipid droplets in these cells. As evidenced by the results of Oil Red O staining performed 8 days post-induction, parental C3H10T1/2 and C3H-EV cells exhibited similar capability in lipid droplet formation, whereas there were more C3H-ZNF521 cells accumulating lipid droplets than parental C3H10T1/2 and C3H-EV cells (Figure 2A). On the other hand, we did not observe spontaneous lipid droplet formation in prolonged cultures of C3H10T1/2, C3H-EV, and C3H-ZNF521 cells maintained in medium without supplement of differentiation inducers (data not shown). Next, we induced C3H-EV and C3H-ZNF521 cells to undergo adipogenic differentiation, and harvested cells 0, 2, 3, 4, 5, 7, and 8 days post-induction to examine the expression of PPARγ2 and two adipocyte markers, aP2 and adiponectin. RT-qPCR analyses showed that PPARγ2 expression in both groups of cells were similar during the first 4 days. In the last 4 days, PPARγ2 expression in both groups of cells increased; however, its level in C3H-ZNF521 cells surpassed that of C3H-EV cells (Figure 2B). Expression of aP2 and adiponectin also increased in both groups of cells during differentiation, but C3H-ZNF521 cells expressed significantly higher levels of expression, especially during the last 2 days, than C3H-EV cells. It was noted that endogenous Zfp521 mRNA levels increased in a similar kinetics in both groups. Subsequently, we performed Western blot analyses to examine PPARγ expression in C3H-EV and C3H-ZNF521 cells harvested 0, 3, 5, and 8 days post-induction. As shown in Figure 2C, PPARγ1 expression was induced intensively in C3H-EV and C3H-ZNF521 cells, while PPARγ2 expression was induced in C3H-EV and C3H-ZNF521 cells with a similar intensity 3 days post-induction. However, PPARγ2 was detected in C3H-ZNF521 but not C3H-EV cells harvested 5 and 8 days post-induction (Figure 2C). Collectively, our data indicated that ZNF521 overexpression was able to increase, selectively, PPARγ2 expression in C3H10T1/2 cells during adipogenic differentiation. It was noted that the expression kinetics of PPARγ2 mRNA were different from those noted in osteoblastic differentiation (Figure 1D), and that overexpression of Zfp521 also promoted adipogenic differentiation of C3H10T1/2 cells (Supplementary Figure S1). Next, we examined if ZNF521 overexpression also affected the expression of two other transcription factor, C/EBPβ and C/EBPδ, which are known to govern the early stages of the transcriptional program of adipogenesis. Western blot analyses showed that ZNF521 overexpression was able to induce the expression of C/EBPδ, but not C/EBPβ, in cells cultured in medium without the supplement of differentiation inducers (Figure 2D).

### ZNF521 transactivated the promoter activity of PPARγ

**Figure 3:**
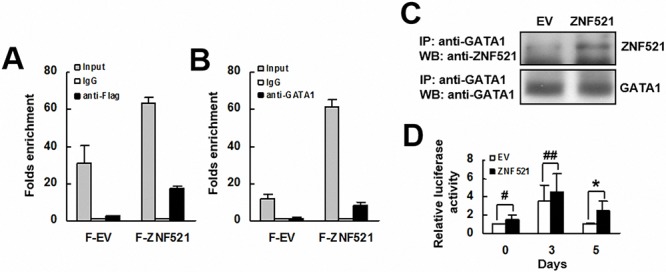
The interaction of ZNF521 with PPARγ promoter **A.** ChIP assays and RT-qPCR analyses. C3H10T1/2 cells expressing flag-tagged ZNF521 (F-ZNF521) or flag-tagged control (F-EV) were subjected to ChIP assays using either anti-Flag and anti-IgG (A) or anti-GATA1 and anti-IgG antibodies **B.**, and then to RT-qPCR analyses. Data represents the mean ± S.D. from three experiments. **C.** Immunoprecipitation plus Western blot analyses. ZNF521-overexpressing (ZNF521) and control (EV) cells were subjected to immunoprecipitation assays, and then Western blot analyses for ZNF521 and GATA1. Representative blots are shown. **D.** Luciferase assays. EV and ZNF521 cells receiving luciferase reporter driven by *PPARγ* promoter were harvested 0, 3, and 5 days after adipogenic induction and were subjected to luciferase assays. Normalized luciferase activities were compared with that of EV cells harvested at day 0 (to which *a* value of 1 was assigned). Data represent the mean ± S.D. from three experiments. *, *P* < 0.01; ^#^, *P* = 0.171; ^##^, *P* = 0.229 versus control.

Next, we investigated how ZNF521 induces the expression of PPARγ2 mRNA. First, we examined if ZNF521 interacted with *PPARγ* promoter. We overexpressed either flag-tagged ZNF521 or flag-tagged control in C3H10T1/2 cells transiently, and performed chromatin immunoprecipitation (ChIP) assays using either an anti-flag or a control antibody and RT-qPCR analyses to measure in the precipitates the levels of DNA fragment containing GATA1-binding sequence derived from *PPARγ* promoter. The results showed that the protein complexes containing flag-tagged ZNF521 bind to *PPARγ* promoter (Figure 3A). We also performed ChIP assays using either an anti-GATA1 or a control antibody and RT-qPCR analyses. The results indicated that the protein complexes containing GATA1 bind to *PPARγ* promoter (Figure 3B). Subsequently, we performed immunoprecipitation assays and Western blot analyses to show the association between ZNF521 and GATA1 in C3H10T1/2 cells (Figure 3C). Then, we examined if ZNF521 overexpression activated mouse *PPARγ* promoter. C3H-EV and C3H-ZNF521 cells were transfected with a plasmid harboring a luciferase reporter under control of the *PPARγ* promoter. These cells were induced to undergo adipogenic differentiation, and were harvested 0, 3, and 5 days post-induction. Subsequent luciferase assays showed that the luciferase activities of C3H-ZNF521 cells harvested 0 and 3 days post-induction were slightly higher than that of corresponding controls, which, however, did not reach statistical significance (Figure 3D). On the other hand, the luciferase activity of C3H-ZNF521 cells harvested 5 days post-induction was approximately 2.5 fold of that of C3H-EV cells (*P* < 0.006).

### ZNF521 overexpression promoted adipogenic differentiation of C3H10T1/2 cells *ex vivo*

**Figure 4:**
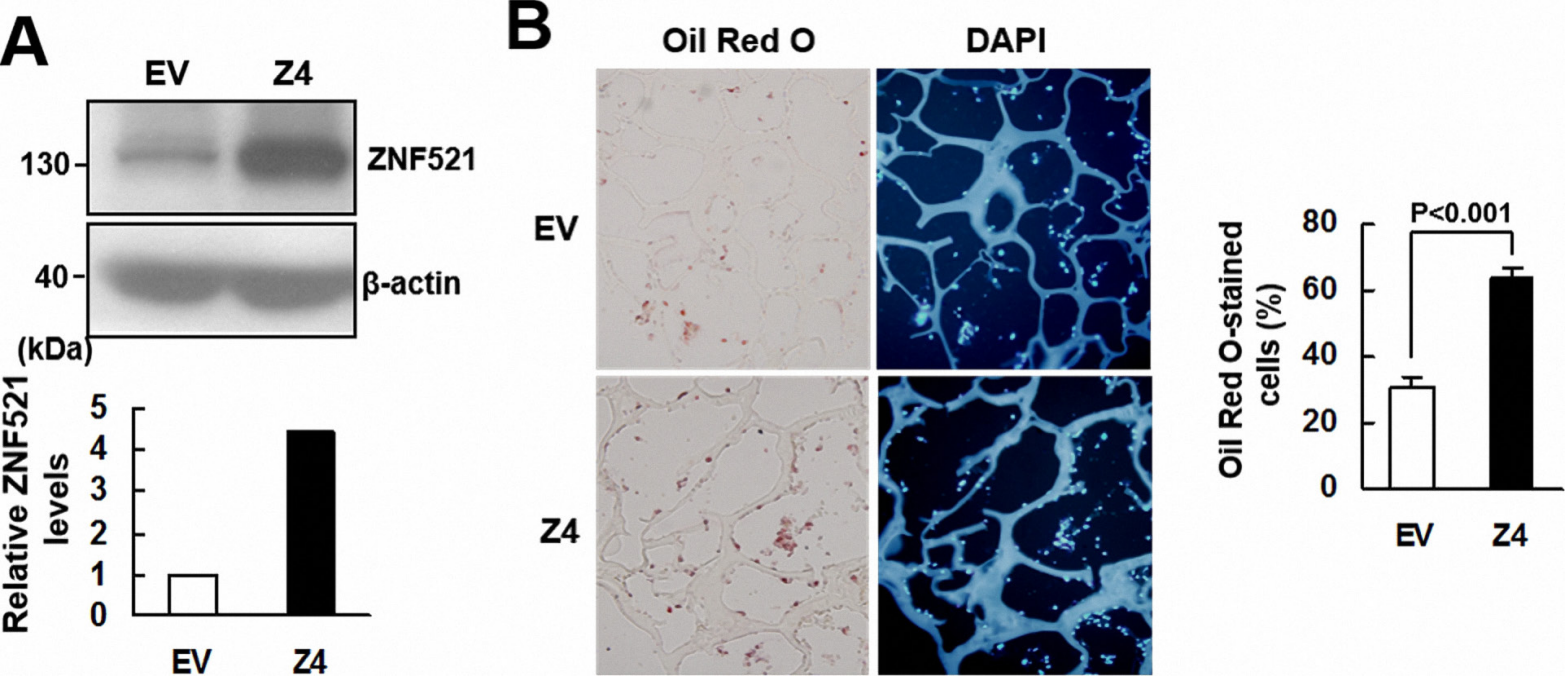
ZNF521 overexpression promoted adipogenic differentiation of C3H10T1/2 cells *ex vivo* **A.** Western blot analyses. ZNF521 overexpression in Z4 cells was measured in relation to control (EV) cells. **B.** *Ex vivo* experiments and histological analyses. Z4 and EV cells were seeded into 3 scaffolds (4 × 10^5^ cells/scaffold) separately, and implanted in pairs into the back of 3 nude mice. Implants were retrieved 2 weeks after implantation and prepared for histological analysis. Representative images are shown. The percentage of Oil Red O-stained cells for each implant was calculated. Data represent the means ± S.D.

To further examine the effect of ZNF521 overexpression to the adipogenic differentiation of C3H10T1/2 cells, we selected from the C3H-ZNF521 cells a Z4 clone. Western blot analyses showed that ZNF521 protein level in Z4 cells is approximately 4.5 fold of that in control cells (Figure 4A). We seeded Z4 and control cells into scaffolds separately, and implanted subcutaneously into nude mice for 2 weeks. We stained the histological sections of retrieved implants with DAPI and Oil Red O to examine if the cells accumulated lipid droplets, and to estimate the percentage of Oil Red O-stained cells in the histological sections of Z4 and control groups. Our data showed that approximately 31% and 64% of DAPI-stained cells in control and Z4 groups, respectively, accumulated lipid droplets (Figure 4B). These results indicated that ZNF521 overexpression promoted adipogenic differentiation of C3H10T1/2 cells *ex vivo*.

### ZNF521-regulated lineage differentiation of human bmMSCs

**Figure 5:**
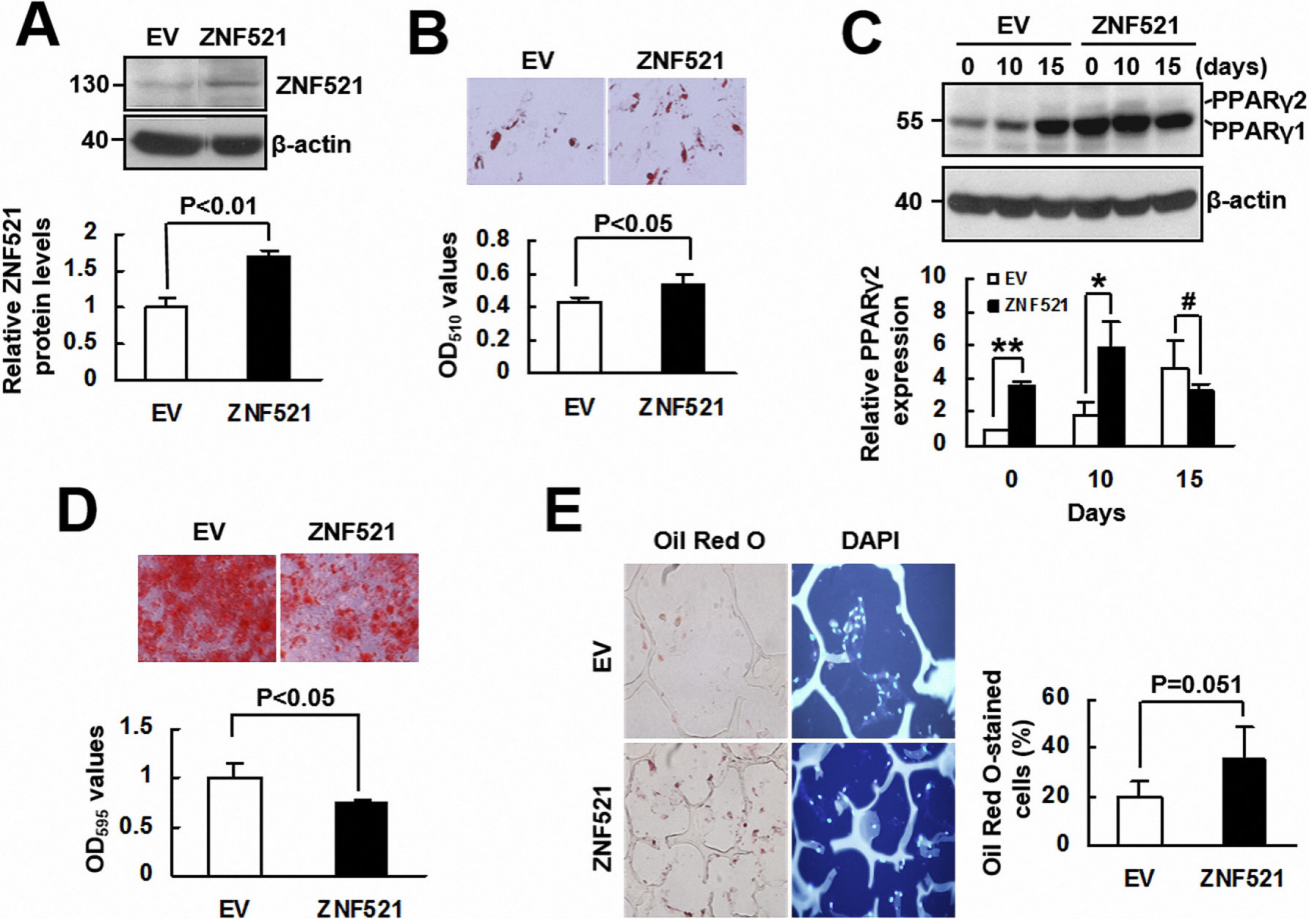
The effect of ZNF521 overexpression to the lineage differentiation of human bmMSCs **A.** Western blot analyses. Relative ZNF521 protein levels of control (EV) and ZNF521-overexpressing (ZNF521) bmMSCs were measured. Data represent the means ± S.D. from three preparations. **B.** Adipogenic induction. EV and ZNF521 cells were induced to undergo adipogenic differentiation and stained with Oil Red O. Representative images are shown. Stains were quantitated. Each value represents the mean ± S.D. of three triplicate experiments. **C.** Western blot analyses. EV and ZNF521 cells as described in (B) were harvested as indicated for the measurement of PPARγ2 protein expression in relative to that of EV control at day 0 (to which *a* value of 1 was assigned). Data represent the means ± S.D. from three experiments. *, *P* < 0.05; **, *P* < 0.001; ^#^, *P* = 0.314. **D.** Osteoblastic induction. EV and ZNF521 cells were induced to undergo osteoblastic differentiation and stained with Alizarin Red S. Representative images are shown. Stains were solubilized and measured at 595 nm. Data represent the means ± S.D. of three triplicate experiments. **E.** *Ex vivo* experiments and histological analyses. EV and ZNF521 cells were seeded into scaffolds (4 × 10^5^ cells/scaffold) separately for *ex vivo* experiments. The percentage of Oil Red O-stained cells was calculated. Representative images are shown. Data represent the means ± S.D.

**Figure 6:**
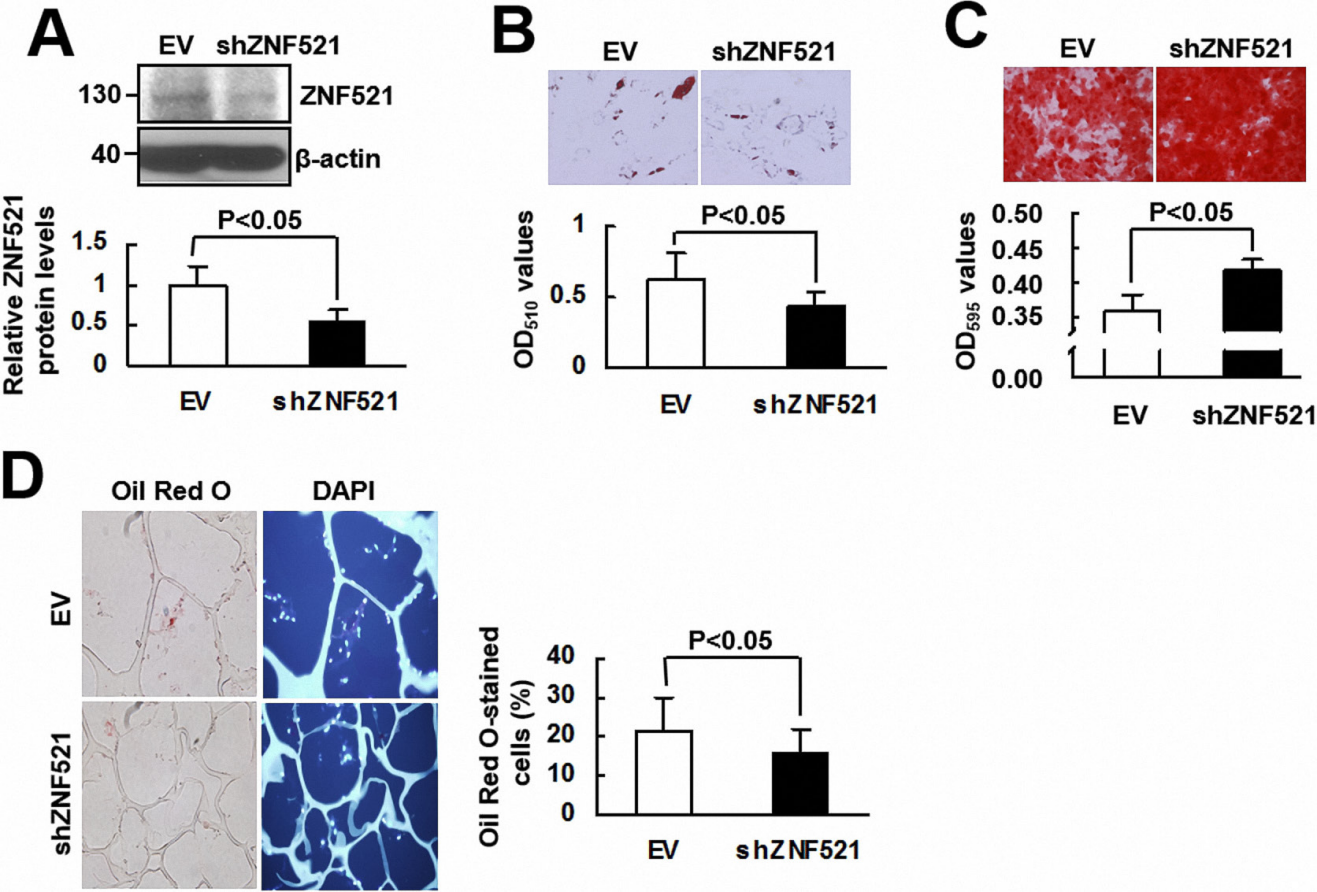
The effect of ZNF521 knockdown on the lineage differentiation of human bmMSCs **A.** Western blot analyses. Relative ZNF521 protein levels of control (EV) and ZNF521-knockdown (shZNF521) bmMSCs were shown. Data represent the means ± S.D. from three preparations. **B.** Adipogenic induction. EV and shZNF521 cells were induced to undergo adipogenic differentiation and were stained with Oil Red O. Representative images are shown. Stains were solubilized and measured at 510 nm. Each value represents the mean ± S.D. of three triplicate experiments. **C.** Osteoblastic induction. EV and shZNF521 cells were induced to undergo osteoblastic differentiation and stained with Alizarin Red S. Representative images are shown. Stains were solubilized and measured at 595 nm. Data represent the means ± S.D. of three triplicate experiments. **D.** *Ex vivo* experiments and histological analyses. EV and shZNF521 cells were seeded into scaffolds (4 × 10^5^ cells/scaffold) separately for *ex vivo* experiments. The percentage of Oil Red O-stained cells was calculated. Representative images are shown. Data represent the means ± S.D.

To investigate whether ZNF521 is able to regulate adipogenic differentiation of human bmMSCs, we overexpressed ZNF521 in bmMSCs (approximately 69% increase) (Figure 5A), and induced cells to undergo adipogenic differentiation. As examined 15 days post-induction, ZNF521-overexpressing bmMSCs accumulated approximately 27% more lipid droplets than control cells did (Figure 5B). Next, we induced ZNF521-overexpressing and control bmMSCs to undergo adipogenic differentiation, and measured PPARγ2 levels in cells harvested 0, 10, and 15 days post-induction. As shown in Figure 5C, PPARγ2 expression was not evident in control cells until 15 days post-induction, whereas it was detected in ZNF521-overexpressing cells at 0, 10, and 15 days post-induction; also, PPARγ2 levels in ZNF521-overexpressing bmMSCs harvested 0 and 10 days post-induction were 3~4 fold of that in corresponding control bmMSCs. Next, we induced ZNF521-overexpressing and control bmMSCs to undergo osteoblastic differentiation for 15 days, and found that ZNF521 overexpression decreased differentiation activity by approximately 25% (Figure 5D). In addition, we seeded ZNF521-overexpressing and control cells into scaffolds separately, and implanted the cells in pair-wise manner into nude mice subcutaneously for 2 weeks. Four pairs of implants were retrieved. Histological examination showed that the percentage of Oil Red O-stained cells from the EV and ZNF521 overexpression groups of the first, second, third, and fourth pair of implants were 13.4% and 45.2%, 19.1% and 31.1%, 17.7% and 17.2%, and 29.9% and 50.2%, respectively. These data indicated that approximately 20% and 35.9% of DAPI-stained control and ZNF521-overexpressing cells, respectively, accumulated lipid droplets (Figure 5E). Statistical analyses showed a moderate significance in the comparison of differentiation activities of control and ZNF521-overexpressing cells (*P* = 0.051). These data indicated that ZNF521 overexpression could enhance adipogenic differentiation of human bmMSCs. On the other hand, we knocked down ZNF521 expression in bmMSCs (approximately 46% decrease) (Figure 6A), and found that it decreased lipid droplet formation by approximately 30% compared with control (Figure 6B). As we induced ZNF521-knockdown and control bmMSCs to undergo osteoblastic differentiation for 15 days, Alizarin Red S staining showed that ZNF521 knockdown increased differentiation activity by approximately 16.4% (Figure 6C). So then, we seeded ZNF521-knockdown and control cells into scaffolds separately, and implanted the cells in pairs into nude mice subcutaneously for 2 weeks. Five pairs of implants were retrieved. Histological examination showed that the percentage of Oil Red O-stained cells from the EV and ZNF521-knockdown groups of the first, second, third, fourth, and fifth pair of implants were 14.2% and 8.4%, 20.7% and 17.7%, 15.6% and 8.1%, 17.1% and 19.1%, and 38% and 23.8%, respectively. These data indicated that approximately 21.1% and 15.4% of DAPI-stained control and ZNF521-knockdown cells, respectively, accumulated lipid droplets (Figure 6D). Statistical analyses showed a significant difference in the differentiation activities between control and ZNF521-overexpressing cells (*P* < 0.05). These data indicated that ZNF521 knockdown could repress adipogenic differentiation of human bmMSCs.

## DISCUSSION

Bone mass reaches its peak around the second or the third decade of life, and declines thereafter with advancing age. Such an age-related bone loss is gender-independent, and is believed to be a critical component in the pathogenesis of osteoporosis. To elucidate the mechanisms mediating age-related bone loss, we have been searching for its intrinsic regulators using human bmMSCs. We assumed that the expression of such regulators in bmMSCs is likely to be age-associated, and that these regulators are involved in regulating the osteoblastic and adipogenic differentiation of bmMSCs. Accordingly, we have previously identified *ZNF521* as an age-associated gene in human bmMSCs, and here we have examined its role in osteoblastic and adipogenic differentiation of mouse C3H10T1/2 cells and human bmMSCs.

Given the findings that ZNF521 expression is up-regulated in human bmMSCs with advancing age [13], and that bone loss is commonly accompanied by decreased osteoblast numbers in aging bones, we at first examined the impact of ZNF521 overexpression on the osteoblastic differentiation of C3H10T1/2 cells. Our data indicated ZNF521 as a suppressor of osteoblastic differentiation by showing the data that (i) osteoblastic induction decreased mouse Zfp521 expression in C3H10T1/2 cells (Figure 1), (ii) ZNF521 overexpression decreased osteoblastic differentiation and expression of Runx2 mRNA in C3H10T1/2 cells (Figure 1), and (iii) ZNF521 overexpression decreased osteoblastic differentiation, whereas ZNF521 knockdown increased osteoblastic differentiation of human bmMSCs (Figures 5 and 6). To address the regulatory role of ZNF521 in osteoblastic differentiation, we showed that ZNF521 overexpression inhibited Runx2 expression but increased PPARγ2 expression as well as lipid droplet formation in C3H10T1/2 cells undergoing osteoblastic differentiation (Figure 1). It has been shown that PPARγ2 overexpression in osteoblastic UAMS-33 cells is able to repress Runx2 expression and convert the cells to adipocytes [16]; also, in a transgenic mouse model, partially knockout of PPARγ enhances bone formation through increased osteoblastogenesis [17]. These findings support PPARγ2 as a suppressor of osteoblastic differentiation. Thus, while ZNF521 overexpression might repress osteoblastic differentiation by inhibiting Runx2, it is tempting to speculate that up-regulation of PPARγ2 expression could be a critical step for ZNF521 to repress osteoblastic differentiation.

On the other hand, our data showed that overexpression of human ZNF521 or mouse Zfp521 enhanced adipogenic differentiation, and that induction of adipogenic differentiation increased Zfp521 mRNA expression in C3H10T1/2 cells (Figure 2 and Figure S1). Our studies further demonstrated the enhancing effect of ZNF521 overexpression (~4.5-fold increase) on the adipogenic differentiation of C3H10T1/2 cells *ex vivo* (Figure 4). In addition, we showed that in human bmMSCs, ZNF521 overexpression promoted adipogenic differentiation *in vitro* and *ex vivo*, but repressed osteoblastic differentiation *in vitro* (Figure 5), whereas ZNF521 knockdown significantly repressed adipogenic differentiation *in vitro* and *ex vivo*, but promotes osteoblastic differentiation *in vitro* (Figure 6). It is worthy to note that for the *ex vivo* experiments as described in Figures 5 and 6, we also stained the histological sections of ZNF521-overexpressing, ZNF521-knockdown, and their corresponding controls with Alizarin Red S. Unfortunately, we could hardly find the stained cells in all groups (data not shown). It is possible that human bmMSCs might not be able to undergo osteoblastic differentiation *ex vivo* as efficiently as *in vitro* in two weeks. Recently, Kang *et al*. reported that induction of adipogenic differentiation decreases mouse Zfp521 mRNA expression in C3H10T1/2 cells [15]. We noted that their experiments were conducted in the presence of rosiglitazone, a PPARγ agonist. Thus, we have also examined the impact of troglitazone, a PPARγ agonist, on the outcomes of our studies. We discovered that in the presence of troglitazone, increased adipogenic differentiation was not necessarily linked to increased Zfp521 mRNA expression in C3H10T1/2 cells (Figure S1). With these results in mind, our data indicated ZNF521 as an enhancer of adipogenic differentiation, and supported the notion that increased ZNF521 expression in bmMSCs might contribute to the development of fatty marrows in aging bones. Interestingly, Kang *et al*. have reported that *Zfp521*^−/−^ embryos exhibit increased adipogenesis [15], which together with our data, imply that ZNF521 might exhibit different effects on adipogenesis in the processes of development and the aging of bones.

Consistent with its enhancing effect on adipogenic differentiation, ZNF521 was able to regulate PPARγ2 expression in C3H10T1/2 cells and human bmMSCs. PPARγ has been recognized as a master regulator of adipogenesis [18]. Our data showed that while differentiation alone induced PPARγ2 expression in control C3H10T1/2 cells, ZNF521 overexpression further enhanced PPARγ2 expression at protein and mRNA levels (Figure 2, 2B and 2C), and similar results are shown in human bmMSCs (Figure 5C). These data suggested that up-regulation of PPARγ2 expression might play an important role in enhancing adipogenic differentiation by ZNF521 overexpression. To address how ZNF521 overexpression up-regulated PPARγ2 expression, we show that (i) ZNF521 overexpression increased *PPARγ* promoter activity in C3H10T1/2 cells undergoing adipogenic differentiation (Figure 3D), and (ii) ZNF521 overexpression alone induced C/EBPδ expression (Figure 2D), a transactivator of *PPARγ* [18, 19] and triggered protein-binding activity on *PPARγ* promoter (Figure 3, 3A and 3B). It seems that ZNF521 overexpression might promote *PPARγ* expression at transcriptional level. It is worthy to note that ZNF521 complexes with GATA1 in C3H10T1/2 cells (Figure 3C). GATA1 is the key regulator of erythroid differentiation. It has been reported that ZNF521 directly interacts with GATA1, inhibits GATA1 activity, and down-regulates erythroid cell differentiation [20]. Our data showed that ZNF521 overexpression increased the binding of GATA1 to its responsive element on *PPARγ* promoter (Figure 3B). The issues regarding whether and how GATA1 is involved in the enhancement of PPARγ2 expression and adipogenesis by ZNF521 overexpression require further investigation.

In summary, we have provided evidences to show that elevated ZNF521 expression is able to enhance adipogenic differentiation while repress osteoblastic differentiation of C3H10T1/2 cells and human bmMSCs. Given our previous finding that ZNF521 expression is up-regulated in human bmMSCs with advancing age, our data suggest that up-regulation of ZNF521 expression in bmMSCs might be a critical part of the mechanisms causing the age-related bone loss. Therefore, elucidating the mechanisms by which ZNF521 is up-regulated and enhances adipogenic differentiation in human bmMSCs is expected to provide important information for the design of therapeutic strategies for counteracting age-related bone loss, and for improving bone formation in aged bones to treat osteoporosis.

## MATERIALS AND METHODS

### Plasmids

Full-length human *ZNF521* or mouse *Zfp521* cDNA was cloned into pcDNA3.1(−) vector (Invitrogen, CA, USA) to generate pcDNA3.1-ZNF521 or pcDNA3.1-Zfp521. A cDNA fragment encoding a 3-repeat-FLAG epitope was cloned 5′ to the ZNF521 cDNA to generate pcDNA3.1–3xFLAG-ZNF521 which expresses FLAG-tagged ZNF521 proteins. A DNA fragment corresponding to the region from −1990 to +8 of *PPARγ* gene was cloned into the Kpn I-Xho I site of pGL3-basic vector (Promega, WI, USA) to generate pGL3-PPARγ-luc. For ZNF521 overexpression in human bmMSCs, human ZNF521 cDNA was cloned into pLAS2w.Pneo vector to generate pLAS2w-ZNF521 for Lentivirus preparation. pLAS2w.Pneo and pshRNA_ZNF521_ (a plasmid harboring shRNA targeting ZNF521 mRNA) were purchased from the National RNAi Core Facility at Academia Sinica, Taiwan.

### Lentivirus preparation and infection

For ZNF521 overexpression or knockdown in human bmMSCs, pLAS2w-ZNF521 or pshRNA_ZNF521_ was cotransfected with gag-pol and VSV-G-expressing plasmids into 293T cells. Viral supernatant was harvested 2 and 3 days after transfection and filtered through 0.45-μm filters. For infection, bmMSCs were infected with virus (MOI = 40) for 3 h in the presence of polybrene (8 μg/ml), and then maintained in regular medium.

### Cell culture

C3H10T1/2 cells were purchased from American Type Culture Collection (ATCC), and were maintained in DMEM medium (GIBCO-BRL, CA, USA). C3H10T1/2 cultures which stably overexpressed ZNF521 or Zfp521 were prepared by transfecting cells with either pcDNA3.1-ZNF521 or pcDNA3.1-Zfp521 using Fugene reagent (Promega, WI, USA), and selected with G418 (1 mg/ml). Antibiotic-resistant cells were pooled and designated as C3H-ZNF521 and C3H-Zfp521, respectively. On the other hand, several clones were selected from C3H-ZNF521 cells, and among them, Z4 clone expressed the highest level of ZNF521 protein. Therefore, Z4 clone was used in *ex vivo* experiments. Human bmMSCs were obtained from a 49-year-old male donor with informed consent. Cells were maintained in low glucose Dulbecco's modified Eagle's medium (GIBCO-BRL, CA, USA). Culture media were supplemented with 10% fetal bovine serum (FBS), glutamine, penicillin, and streptomycin. Cells were cultured at 37 °C in a humidified atmosphere containing 5% CO_2_.

### Induction of adipogenic and osteoblastic differentiation of C3H10T1/2 cells and human bmMSCs

For adipogenic differentiation of C3H10T1/2 cells, confluent cultures were maintained in regular medium plus 1 μM dexamethasone, 0.5 μM isobutylmehtylxanthine, and 10 μg/ml insulin for 3 days, then in regular medium plus 10 μg/ml insulin for 2 days. Cells were then maintained in regular medium until the end of the experiment. For osteoblastic differentiation of C3H10T1/2 cells, confluent cultures were maintained in regular medium plus 0.1 μM dexamethasone, 0.2 mM ascorbic acid 2-phosphate, and 10 mM glycerol 2-phosphate until the end of the experiment, with medium changed every three days. For adipogenic differentiation of human bmMSCs, cells were cultured in regular medium plus 1 μM dexamethasone, 0.5 μM isobutylmehtylxanthine, and 10 μg/ml insulin for 3 days and then in regular medium plus 10 μg/ml insulin for 2 days repeatedly until the end of the experiment. Induction of osteoblastic differentiation of human bmMSCs was performed as described for that of C3H10T1/2 cells. At the end of experiments, cells were fixed and stained by Oil Red O (Sigma-Aldrich, MO, USA) for adipogenic differentiation, or by Alizarin Red S (Sigma-Aldrich, MO, USA) for osteoblastic differentiation.

### Quantitative real-time PCR (RT-qPCR) and Western blot analyses

Total RNA was isolated from cells using Trizol (Life Technologies, CA, USA) and subjected to reverse transcription-PCRs to generate complimentary DNAs. RT-qPCR was performed using the Power SYBR Green PCR Master Mix (Applied Biosystems, CA, USA). The 5′ and 3′ primers used were as follows: human ZNF521, CAACGTGTGCTCTCGAACCTT and GCCTA GGTGGGTCTGCATATG; mouse Zfp521, GAAAC CGAGATCCCTCAAAGA and TCTGGCCTCTTCTT GCAGTC; mouse PPARγ2, TCGCTGATGCACTGC CTATG and GAGAGGTCCACAGAGCTGATT; mouse aP2, AAGAGAAAACGAGATGGTGACAA and CTTGTG GAAGTCACGCCTTT; mouse adiponectin, AGCCGCT TATATGTATCGCTCA and TGCCGTCATAATGATTC TGTTGG; mouse Runx2, CTCCGCTGTGAAAAACC and TGAAACTCTTGCCTCGTCC; mouse osteopontin, CCATCTCAGAAGCAGAATCTCC and ATGGT CATCA TCGTCGTCC; and mouse β-actin, CCCT GGCA CCCAGCAC and GCCGATCCACACGGAGTAC. All RT-qPCRs were performed in triplicate on an ABI PRISM 7000 Sequence Detector System. The relative mRNA levels were calculated using the 2^−△△CT^ method, with β-actin mRNA as a normalizer. For Western blot analysis, aliquots (40 μg) of whole-cell lysates were separated on 10% SDS-polyacrylamide gels, and electrotransferred onto polyvinylidene membranes. The membranes were incubated with anti-ZNF521, anti-PPARγ2 (Santa Cruz, CA, USA), and anti-β-actin (BD Biosciences, CA, USA) antibodies, and the signals were obtained by enhanced chemilluminescence (PIERCE, IL, USA).

### Immunoprecipitation assay

To examine the association between ZNF521 and GATA1, C3H10T1/2 cells (1 × 10^6^) were transfected with either 8 μg of pcDNA3.1-ZNF521 or equal molar of empty pcDNA3.1. Cells were lyzed in RIPA buffer 40 h post-transfection, and incubated with mouse preimmune serum plus protein A/G-agarose at 4°C for 16 h with gentle shaking. The supernatants were collected. Each lysate (200 μg) was then immunoprecipitated with 2 μg of anti-GATA1 antibody plus protein A/G-agarose at 4°C for 16 h. After low-speed centrifugation, the supernatants were discarded. The remaining agarose beads were washed 4 times with lysis buffer, and then subjected to Western blot analysis using anti-ZNF521 and anti-GATA1 (Cell Signaling, MA, USA) antibodies.

### Chromatin immunoprecipitation (ChIP) and luciferase assay

For ChIP assay, C3H10T1/2 cells (1 × 10^6^) were transfected with either 8 μg of pcDNA3.1–3xFLAG-ZNF521 (F-ZNF521) or equal molar of empty pcDNA3.1–3xFLAG (F-EV). Cells were lyzed 40 h post-transfection, and equal amounts of lysates were subjected to sonication on ice to break down the chromatins into small fragments, and then mixed with antibodies as indicated for 16 h, and then mixed with protein A beads for 4 h. Beads were washed and collected by centrifugation. RNAs were isolated from the precipitated ribonucleoprotein complexes and subjected to RT-qPCR analyses using the 5′ and 3′ primers as GCTAGACCAGCCCCCACTTT and AGGCGAGGCTGGAATTGG, respectively. The PCR product contains a GATA1-binding sequence. To examine the influence of ZNF521 overexpression on the activity of *PPARγ* promoter, ZNF521-overexpressing and control C3H10T1/2 cells (1 × 10^6^) were transfected with 8 μg pGL3-PPARγ-luc together with a Renilla luciferase reporter (0.1 μg) using the Effectene reagent (Qiagen, CA, USA). Cells were then induced to undergo adipogenic differentiation, and were harvested at the times indicated for luciferase assays using the Dual-Luciferase Reporter Assay System (Promega, WI, USA).

### *Ex vivo* experiments and histological analysis

Male nude mice (8 weeks old) were purchased from the National Laboratory Animal Center in Taiwan. Cells were seeded into 2 mm × 4 mm × 4 mm gelatin/EDC scaffolds (a kind gift from Dr. Feng-Huei Lin, NHRI, Taiwan), and implanted subcutaneously into the back of nude mice. Animals were housed with a cycle of 12 hours of light and 12 hours of dark, and fed *ad libitum*. Implants were retrieved at the time as indicated and prepared for histological analysis. Histological sections were prepared from each implant and stained with Oil Red O and DAPI. For each implant, the numbers of Oil Red O-stained cells out of 150 DAPI-stained cells were counted, and the percentage of Oil Red O-stained cells was calculated.

### Statistical analyses

Statistical difference was determined using Student's *t* test. Paired *t* test was used in *ex vivo* experiments.

## SUPPLEMENTARY FIGURE


