# Supplementary material for: Zinc finger factor 521 enhances adipogenic differentiation of mouse multipotent cells and human bone marrow mesenchymal stem cells
Source: Oncotarget. 2015 May 6;6(17):14874–84. doi: 10.18632/oncotarget.3900 (PMC4558122; doi:10.18632/oncotarget.3900)
Supplement: Supplementary file 1 [file oncotarget-06-14874-s001.pdf]

SUPPLEMENTARY FIGURE

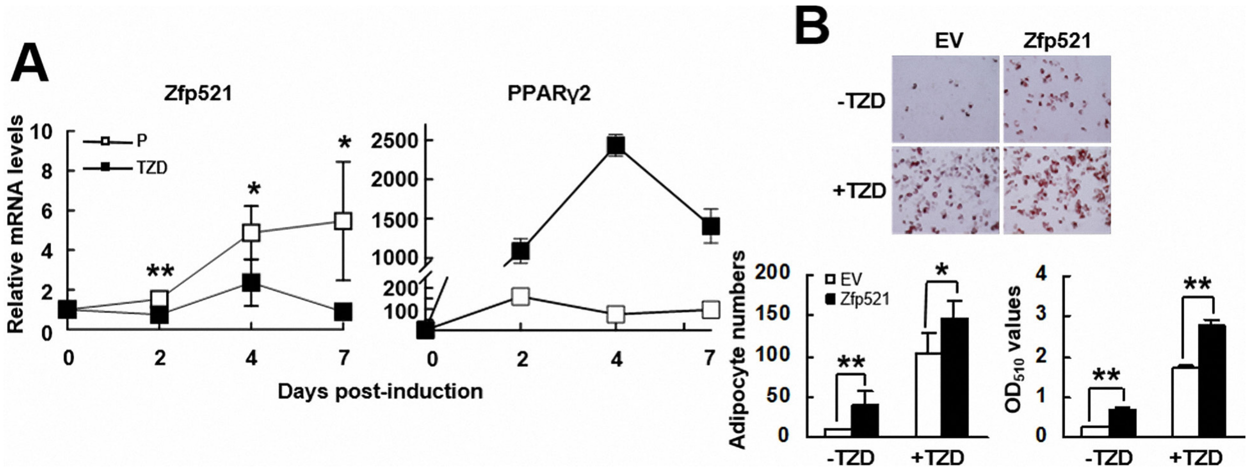

Supplementary Figure S1: The effect of mouse Zfp521 overexpression on the adipogenic differentiation of C3H10T1/2 cells. A. RT-qPCR analyses. C3H10T1/2 cells were induced to undergo adipogenic differentiation without troglitazone (P) or with troglitazone (TZD, 5 μM) co-treatment. RNAs were isolated from cells harvested 0, 2, 4, and 7 days post-induction, and subjected to RT-qPCR analyses for the levels of Zfp521, PPARγ2, and β-actin mRNAs. Relative expression levels were calculated by comparing the normalized values of day 2, 4, and 7 to that of day 0 (to which *a* value of 1 was assigned). Data represent the means ± S.D. from three independent experiments. *, $P < 0.05$; **, $P < 0.005$ compared to corresponding controls. **B.** Adipogenic induction. Confluent cultures of C3H-EV (EV) and C3H-Zfp521 (Zfp521) cells were induced to undergo adipogenic differentiation with or without TZD co-treatment. Cells were fixed and stained with Oil Red O 8 days post-induction. Representative images are shown. Stained cells were counted in 6 fields under a 200X high-power field. Each value represents the mean ± S.D. of 18 counts (left). Then, Oil Red O stains were solubilized and measured at 510 nm (right). *, $P < 0.05$; **, $P < 0.005$ compared to corresponding controls. The results showed that Zfp521 mRNA levels increased during differentiation (Figure S1A). Interestingly, concomitant treatment of cells with troglitazone, a PPARγ agonist, inhibited the induction of Zfp521 expression during differentiation. Oil Red O staining performed 8 days post-induction showed that Zfp521 overexpression increased lipid droplet formation in C3H10T1/2 cells, and troglitazone further potentiated lipid droplet formation (Figure S1B). Taken together, the results indicated that like ZNF521, Zfp521 might play as a positive regulator in the adipogenic differentiation of C3H10T1/2 cells, and that TZD might link the induction of PPARγ2 expression and adipogenic differentiation of C3H10T1/2 cells to a decrease of Zfp521 expression.
